# Supplementary material for: Are carbon emissions trading and green financial instruments synergistic? -Comprehensive quantitative research based on content analysis
Source: PLoS One. 2024 Mar 7;19(3):e0298601. doi: 10.1371/journal.pone.0298601 (PMC10919601; doi:10.1371/journal.pone.0298601)
Supplement: S1 Data — (ZIP) [file pone.0298601.s001.zip › Data/地级市Z_F工作报告.zip!/╣π╢1⁄2/╜╥╤⌠╩╨/└┤╫╘╣1⁄2╓┌║┼í╛┬φ┐╦╩2╛▌═°í┐_2016.docx]

各位代表：

现在，我代表市人民政府向大会作工作报告，请予审议，并请市政协各位委员和其他列席人员提出意见。

“十二五”时期工作回顾

“十二五”时期，市政府在省委省政府和市委的正确领导下，在市人大及其常委会、市政协的监督支持下，全面贯彻党的十八大和十八届三中、四中、五中全会及习近平总书记系列重要讲话精神，主动适应经济发展新常态，深刻把握五大发展理念，积极探索供给侧结构性改革，认真落实振兴发展和创新驱动两大发展战略，按照“抓落实、促发展，抓基层、强基础”的部署，践行“强化效率、彰显公平、惠民执政、绿色发展”施政理念，着力产业强市，强化对德合作，实施“互联网＋”，推进绿色发展，积极应对困难和挑战，推动全市经济社会持续健康发展。

——经济发展稳中提质。2015年生产总值1890亿元，五年（下同）年均增长11.8%；规模以上工业增加值1123亿元，年均增长19.5%；固定资产投资1362亿元，年均增长28.6%；社会消费品零售总额857亿元，年均增长17.4%；外贸进出口70.44亿美元，年均增长14.2%；地方公共财政预算收入77.4亿元，年均增长14.9%；城乡居民人均可支配收入16308元，年均增长12.3%；金融各项存款余额1837.87亿元，贷款余额931.72亿元，分别比2010年增长90.8%和132.5%，贷存比达到50.7%，比2010年提高9.1个百分点；单位生产总值能耗五年累计下降19.1%。

——“三大建设”初显成效。交通基础设施建设五年累计投资209.16亿元，揭阳潮汕国际机场建成通航；惠来2个10万吨级泊位竣工使用；厦深高铁运行，梅汕客专揭阳段开工；汕湛高速揭博段通车，潮惠、揭惠高速揭阳段加快建设；改造国、省道215公里，新改建地方公路1950公里。中德金属生态城成为全国2个中德中小企业合作区之一；空港经济区、大南海石化工业区、揭阳产业转移园等主题产业平台规划建设扎实推进。中心城区扩容提质步伐加快，揭东获准撤县设区，发行城投债16亿元，省粤东西北股权基金9亿元投入新区建设，国家政策性融资15亿元启动市区棚户区改造。

——产业强市成为共识。实施“6166”计划和产业引进、产业登高工程，每年保障50%以上土地指标用于工业项目，形成“一县一主题”产业发展雏形。实施技改项目928个，完成技改投资438.84亿元，比“十一五”时期增长4.3倍。建设省金融科技产业融合创新综合试验区，引进金融机构18家，设立普惠金融服务中心5个；建成中药材、石化产品2个省级重大交易平台，发布国家首个中药材价格指数。新增国家级质检中心2个，列入国家技术创新示范企业1家、省战略性新兴产业骨干企业3家、高新技术企业51家；新设院士工作站6个，新增国家级企业技术中心2个、省级企业技术中心13个、省级工程技术研发中心23个。新增国家4A级景区2家。举办玉文化节、衬衣节、中药材交易会、电商年货节等，荣获“中国塑料时尚鞋之都”称号。

——对德合作形成格局。开展以德国为重点的国际合作，实施引进型创新。创建全国首个德国工业园区参与规划设计管理营运的园区。承办中国共产党与德国社民党第二次可持续发展对话会。创立中德中小企业合作交流会，组建对德（欧）“百人团”，在德国、奥地利、西班牙创建8个办事处，与德国执政党基民盟经济委员会、德国工商大会、德国雇主协会等9大协会和弗劳恩霍夫研究所、埃斯林根应用科技大学等科教机构建立合作联盟。中德资源再生基地、德国先进技术合作中心、电镀“零排放”项目在中德两国领导人见证下签约，保库智能管网等26家企业落户或达成协议，中德金属生态城成为中德经济合作委员会成员。

——“互联网＋”先行先试。创建国家电子商务示范城市和工业电子商务区域试点城市，建成全国闻名的军埔电商村。2015年全市快递业务量比上年增长197.6%，增速全省第一。实施十万电商人才大培训。启动电商下乡工程，揭西、揭东实现农村电商全覆盖，普宁跻身全国淘宝村集群四强。

——“三农”工作得到加强。被定为全省唯一的“一镇一品”工程示范市，普宁高埔镇、揭东埔田镇和洪阳宝镜院村成为国家“一镇一品”示范镇（村）。抗击百年不遇超强台风和特大洪涝灾害。投入水利资金48.39亿元，完成普宁白坑湖水库综合整治等593宗重点水利工程。启动省级新农村连片示范工程项目52宗，落实美丽乡村“三大工程”建设项目819项。实施“农村清洁工程”，建成69座镇生活垃圾转运站和3410个自然村垃圾收集点。落实扶贫“双到”资金11.42亿元，扶持贫困村项目4448个、贫困户项目13.04万个，帮助脱贫6.3万人。

——环境整治力度加大。建成“零排放”电镀产业园，榕江基本实现无电镀污染。新建成污水处理设施6座，新增日处理能力13.15万吨。依法查处环保违法行为，关停非法电镀加工企业232家、土轧钢企业55家、漂染企业142家，清理整治普宁电子废物经营户942户。2015年，城市空气质量达标率为93.1%，榕江11个常规监测断面有4个水质提升一个类别。“人树一体”理念渐入人心，新增森林碳汇造林116万亩，建设生态景观林带421公里，新增森林绿地面积17.8万亩，森林覆盖率提高至55.39%。揭西县获批国家级生态保护与建设示范区。

——社会民生持续改善。五年教育总投资297.6亿元，建成标准化义务教育学校1424所、规范化幼儿园615所，改造提升薄弱普通高中20所，教育创强取得明显成效，在粤东地区率先实现“3个100%”（100%的镇<街>通过省教育强镇督导验收、100%的县<市、区>通过省教育强县督导验收、100%的县<市、区>通过义务教育发展基本均衡县国家级督导认定）。医改工作稳步推进，创建“三甲医院”3家，康美医院等一批卫生设施建成投用，生育政策改革得到落实。城乡居民基本养老保险和基本医疗保险均实现全覆盖，社会保障体系进一步完善。新增国家级文物保护单位3处，市图书馆建成对外开放，市档案馆晋升为国家二级档案馆。创编潮剧《烽火揭阳》等优秀剧目，“乡乡乐起来”等群众文化活动深入开展。双拥共建更加紧密，阳光征兵经验在全省推广。办好民生实事，为全市人民统一购买政府民生综合责任险，为70岁以上老人投保意外伤害险，为百岁老人、本科以上贫困大学生、乡镇卫生院全日制本科学历以上医生发放津贴补助，免除公立幼儿园在园低保户儿童保教费，组建公安辅警队伍和抢险轻舟队，引入新能源公交公司。实施“一村（社区）一法律顾问”工程，社会治安和信访维稳得到强化，生产安全、消防安全、食品安全形势稳定好转。

——自身建设有效加强。深入开展党的群众路线和“三严三实”教育实践活动，严肃整治“四风”和不严不实问题。人大代表、政协委员建议提案办理满意率均达到100%。市、县两级政务服务中心和网上办事大厅投入使用，建成四级政务服务体系，揭东区作为全省试点在全市率先实现基层公共服务平台建设全覆盖。完成新一轮政府机构改革，推进商事登记和行政审批制度改革，取消、下放行政审批事项440项。市政府系统议事协调机构撤销49%，公务用车制度改革基本完成。成立市人民政府行政复议委员会，强化行政监察和审计监督，廉政建设得到加强。

各位代表！五年的实践，我们深刻体会到：谋划发展，必须主动适应经济发展新常态，必须把握五大发展理念，必须争取地方发展纳入国家战略规划，融入全球资源配置链条；经济工作，应基于创新驱动，源于自然基础，顺于发展态势，善于资源配置，专于久久为功，胜于以实求成。

各位代表！我们在“十二五”期间取得的发展成效，是党中央国务院、省委省政府和市委正确领导的结果，是全市广大干部群众团结拼搏的结果。在此，我代表市人民政府，向全市干部群众，向驻揭解放军和武警部队官兵，向关心支持揭阳改革发展的各界朋友、港澳台同胞、海外侨胞和国际友人表示衷心感谢并致以崇高敬意！

同时，我们也清醒地认识到，当前存在的困难和问题有：思想观念与新常态要求仍有差距；创新驱动能力不足；人才短板突出；转型升级十分紧迫；节能减排任务繁重；民生事业欠账较多，等等。对此，我们将高度重视，在振兴发展进程中加以解决。

“十三五”时期奋斗目标和主要任务

“十三五”时期是全面建成小康社会的决胜阶段，是揭阳实现振兴发展的关键时期。根据市委关于“十三五”规划的建议，市政府编制了《揭阳市国民经济和社会发展第十三个五年规划纲要（草案）》，提请大会审议。“十三五”时期政府工作指导思想是：全面贯彻党的十八大和十八届三中、四中、五中全会及习近平总书记系列重要讲话精神，按照“五位一体”总体布局和“四个全面”战略布局，牢固树立创新、协调、绿色、开放、共享五大发展理念，适应和引领经济发展新常态，着力加强供给侧结构性改革，深入实施振兴发展和创新驱动发展“两大战略”，全面推进“抓落实、促发展，抓基层、强基础”两项部署，以产业强市夯实发展基础，以对德合作实现引进创新，以“互联网＋”彰显后发优势，以绿色生态增强可持续发展能力，努力推动经济增长保持中高速，奋力开创揭阳振兴发展新局面。

“十三五”时期我市经济社会发展主要目标是：生产总值年均增长10%以上；地方公共财政预算收入年均增长11%；城乡居民人均可支配收入年均增长11%；经济社会保持较快健康发展，产业发展迈向中高端，社会事业全面进步，生态环境持续改善，全面深化改革和全面依法治市取得实质性成果，确保与全国同步全面建成小康社会，为基本实现社会主义现代化打下坚实基础。

实现“十三五”规划目标，必须重点抓好几方面：

（一）以改革创新激发发展活力。创新驱动是国家命运所系、世界大势所趋、发展形势所迫。抓创新就是抓发展，谋创新就是谋未来。把创新驱动发展作为核心战略和总抓手，坚定不移走创新立市、创新兴市之路，破除一切制约创新的思想障碍和制度藩篱，让创新活力竞相迸发，让创新源泉充分涌动，使创新成为揭阳发展的引擎。围绕供给侧结构性改革，突出抓好对德合作，实现引进型创新，闯出传统产业转型升级和新兴产业培育壮大“双轨道”。下大力气创造条件引进扶持创新人才，着力建设中德工业4.0研究院、中德创新港孵化器、中德先进技术合作中心、中小微企业孵化器及众创空间等创新平台，大力发展高新技术企业。做强做大中国电商好讲师邀请大赛、电商人才擂台大赛等活动项目，打造创新群体、创新资源与创新消费市场有机对接的有效平台，聚集承载创新全要素，建设创新驱动高地。充分发挥市场对资源配置的决定性作用，更好发挥政府作用。深化行政体制改革，转变政府职能，强化政务效能。深化商事制度改革，营造市场化国际化法治化发展环境。

（二）以均衡协调提升发展水平。按照“一县一产业主题、一重点平台、一产业集群、一产业链条、一产业政策、一产业会展、一产业品牌”的产业发展思路，以“钉钉子”精神，聚精会神，集聚资源，持之以恒地发展县域经济，促进区域经济协调均衡发展。榕城区围绕服务业供给侧改革，在城市化进程中优化服务业布局，发展生活性服务业和生产性服务业，培育互联网＋街区经济、古城文化旅游产业、后汽车产业，推动时尚鞋业从生产端向设计营销双高端转型，建设榕江服务业发展带。揭东区着力建设军埔电商产业园，以面向全国的免费电商人才培训基地建设，打造有全国影响力的电商人力资源集聚地，以互联网＋制造业为切入，建设传统产业转型升级集聚区，规划开发高铁经济区。蓝城区加快建设海峡食品药品合作区，推动玉文化产业转型升级，创建现代物流产业园区。空港经济区要承担起中德中小企业合作区和汕潮揭临港空铁经济合作区揭阳片区的建设任务，建设中德合作先进制造业基地及德国中小企业商品交易平台，同时探索建设玩具、模具、家具、石材供给侧改革试验区。普宁市要自我加压，建设练江绿色产业经济带，沿江布局纺织服装转型升级区、大健康产业带、互联网＋商贸产业链。揭西县要坚守生态底线，发展生态旅游文化产业、互联网＋生态农业及新型电线电缆产业。惠来县着力海洋经济，发展农海产品加工业、临港工业和滨海旅游业。大南海石化工业区要把握石化产业转型升级的倒逼契机，创建石化产业供给侧改革合作区。普侨区、大南山侨区着力侨民职业技能建设，发展互联网＋生态农业、观光农业、健康养老产业。

（三）以绿色生态实现永续发展。深刻吸取牺牲环境生态换取阶段性发展的教训，痛定思痛，坚持经济发展与环境保护并重，严守生态红线，实现绿色发展。与德国莱茵河治理委员会等环保机构深度合作，治理电镀、酸洗、漂染三大污染“病灶”，推进榕江、练江治理与龙江保护，实施大气污染综合治理。深化“人树一体”理念，坚持不懈植树造林，建设榕江、练江、龙江绿色廊道，推进城市加绿、乡村添绿。构建中德合作、国际领先的环保产业。以“一镇一品”为抓手，以临空农业为切入，以观光农业为载体，发展现代生态农业，扎扎实实构建永续发展的绿色家园。

（四）以开放合作增创发展优势。依托国家“一带一路”战略，以中德金属生态城为起点，建设通往德国的“高速公路”，把揭阳打造成为广东对德合作的门户、中国对德合作的枢纽，提升揭阳国际化水平，强化揭阳在全球资源链中的配置能力。举全市之力建设中德中小企业合作区，以对德合作“百人团”及德语夜校建设，增强民众对德合作主动性。扩展对德合作广度，挖掘对德合作深度，实现从产业合作到多领域合作。依托国际航空口岸潮汕机场，强化揭阳在“一带一路”中的节点地位。提升空港、高铁、海港、河港、高速五位一体立体交通体系的功能作用，形成开放格局中的枢纽优势。借鉴吸纳自贸区的经验做法，与汕头海关共建关地合作区，塑造揭阳对外开放合作的体制机制优势。在汕潮揭城市群及汕潮揭临港空铁经济合作区规划建设中主动作为，增创揭阳的辐射支撑作用。重塑新时期“红头船”精神，让揭阳在“一带一路”的闯海弄潮中再铸辉煌。

（五）以共建共享释放发展红利。发展为了人民，必须始终把保障和改善民生作为发展的出发点和归宿点。深刻把握人民群众的根本性、普遍性、迫切性诉求，加大财政民生投入，完善基本公共服务体系建设，促进公共服务均等化。出台更加积极的就业政策，努力实现充分就业。完善社会保障体系，提升社会保障水平。推进教育现代化。建设卫生强市。大力弘扬社会主义核心价值观，繁荣发展文化事业，健全各类公共文体设施，满足群众文体需求。开展精准扶贫，坚决打赢脱贫攻坚战。加强社会管理，确保社会和谐稳定。切实控制地价房价，减轻企业负担，降低城乡生产、生活成本，增强发展成本优势。大力发展民生事业，使发展成果惠及全体人民，让人民群众有更多获得感。

2016年工作安排

2016年是实施“十三五”规划的开局之年，也是本届政府工作的收官之年，承前启后，意义重大。今年经济社会发展主要预期目标是：生产总值增长9%-9.5%；固定资产投资增长11%；社会消费品零售总额增长13%；外贸进出口增长10%；地方公共财政预算收入增长10.5%；城乡居民人均可支配收入增长10%；居民消费价格涨幅控制在3%左右；城镇登记失业率控制在3.5%以内；节能减排约束性指标完成省下达年度计划。

为实现上述目标，我们将重点做好十个方面工作：

（一）下真功夫建设产业强市

强化产业组织化建设。深化“6166”计划，加强行业协会建设，以产业组织化实现产业队伍合作共赢、产业资源聚拢放大、产业链条紧凑共享、产业创新“众建众创”、产业品牌的辐射影响、产业文化根深叶茂。重点推动市重点产业协会与德国产业协会合作，引入借鉴治理模式及协会文化，实现协会效能提升。同时，大力扶持发展产业技术研究院所、产业创新孵化器、产业发展基金、产业会展。

推进产业引进计划。将2016年确定为产业引进年，各县（市、区）要确定产业引进主题，成立引进机构，组织专职和兼职引进队伍，锁定引进目标，制定引进方案，出台引进政策，建设引进平台，扎扎实实开展产业引进工作。

实施产业登高工程。实施百名产业领军人物登高培训、百企对德合作登高、百企科技攻坚、百项金融专项助推百企登高、百企“互联网＋”团队建设及百企品牌提升推广等6项计划，扎扎实实推进产业登高工程。

（二）久久为功深化对德合作

务实建设合作平台。落实中德中小企业合作区起步区建设的政策措施，争取省政府年内出台专项扶持政策，加快中德金属生态城建设。重点建设中德合作“零排放”电镀园区、德国先进技术合作中心、德国先进装备国产化中心，探索轻资本的制造业创办模式。建设中德大道机场连接线，启动广梅汕铁路中德货运站、榕江中德港规划建设。联合揭阳潮汕国际机场、中外运，开辟“揭泰欧”空中、“揭港欧”海上新丝路。联合德国工商大会、埃斯林根市等，打造“互联网＋”、商贸、旅游和文化一体的德贸中心。

办实办活合作载体。落实第五次中德中小企业合作政策磋商会、中国共产党与德国联盟党“中国制造2025”和“德国工业4.0”对话会成果，打造中德中小企业合作政策策源地和中德工业4.0合作试验区。承建首个在德“中国中心”。建设中德中小企业家合作培训基地，承办中德中小企业家合作培训项目。创建德国退休工程师中心，引进德国退休工程师。组织“百人团”走出去、请进来，实现百家企业转型升级的先行先试，以引进创新促进供给侧结构性改革。办好第二届中德中小企业合作交流会，做大做强中德（欧）购物节，实现对德合作载体化、项目化、时效化。

大力推动“中德＋”。把对德合作作为推进供给侧结构性改革、提升揭阳国际化水平的重要抓手，启动“中德＋”，推动中德＋制造业、中德＋商贸、中德＋金融、中德＋教育、中德＋旅游、中德＋文化、中德＋农业，实现对德合作的多领域拓展。举办中德文化艺术节，推动潮汕文化与德国先进文化互鉴发展。强化制度、文化保障，使揭德合作从“一见钟情”到“白头偕老”。

（三）加快推进“互联网＋”行动

抢占电子商务制高点。以电子商务为主抓手，全面实施“互联网＋”。切实抓好“一基地、两大赛、三工程”：打造面向全国的电商免费培训基地；举办中国电商好讲师邀请大赛和中国电商人才擂台大赛，努力形成全国电商人才“孔雀揭阳飞”的局面；推进电商下乡、电商进厂、跨境电商三项工程。实施电商下乡工程，实现电商服务站农村全覆盖，推动互联网＋“一镇一品”。实施电商进厂工程，引导企业上网触电，以“互联网＋”助推企业供给侧改革。实施跨境电商工程，结合德贸中心和关地合作，支持十万电商青年人才零成本创建特色德国网店，让德国中小企业零人力资源成本拥有中国跨境网店，建设德（欧）名优产品线上线下交易平台。

下好智能制造先手棋。联合工信部赛迪研究院、德国创新实验室、德国弗劳恩霍夫研究所创建工业4.0研究院，举办工业4.0培训班，建设工业4.0示范工厂，培育工业4.0企业集群，创建中德工业4.0合作试验区。以巨轮、康美、保库、柏堡龙等为示范，优选产业“两化”融合试点企业，推进“两化”融合贯标试点，鼓励企业引进自动化、数字化、网络化、智能化等先进制造技术，支持优势传统企业设备更新和升级换代，推进产业链、价值链整体提升。

拓展“互联网＋”应用领域。开展互联网宣讲体验活动，增强全社会互联网意识，建设“互联网＋”先行市。联合中国教科院共建“互联网＋”教育示范市，试点开展“云学校”改造建设。推进医疗信息化建设，创立“互联网＋”医院。推进互联网＋政务服务，建设统一的便民服务平台。鼓励以互联网＋创业创新，建设“互联网＋”示范街区，促进互联网和经济社会融合发展。

（四）稳步强化城市建设管理

完善城市规划体系。加强城市规划修编工作，以新理念、新思维抓好城市总体规划修改和汕潮揭临港空铁经济合作区规划及城市交通网络、市政道路、地下管廊、城市公共服务体系、生态环保等各项规划，优化城市空间发展布局。探索实行规划地块绿地面积约束指标，形成嵌入式城市“绿网”。注重城市立面和开放空间设计，提升城市通透性和微循环能力。

优化城市发展格局。加快新城规划建设，带动老城改造提质，在城市建设的同时为未来发展预留空间。推进望江北路、空港大道、国道206线市区段等一批重点市政道路改造建设。对全市公园建设进行统一规划，以市区七大公园建设提升为重点，统筹推进全市公园建设。建设地下管廊示范工程，发展“海绵城市”。以中德中小企业合作区建设拓展城市空间，推动临港空铁经济合作区与中德金属生态城融合开发、并联发展，建设产城融合、产城一体的国际化新城。加强古城保护开发，推进棚户区改造，培育互联网＋街区经济，形成现代与历史融合辉映的特色城市。

提升城市管理水平。推进城市管理体制改革，明确市、区权责，强化城市管理和执法工作。妥善处理城市“三旧”改造项目历史遗留问题，加快商品房“去库存化”。依法推进小产权房清理。强化城市规划控制和立面管理。开展城市环境综合整治，改善市容市貌。启动省文明城市创建工作，支持社区群众参与城市管理，实现城市共治共管、共建共享。

（五）着力构建立体交通体系

完善综合交通网络。投资56亿元，加快交通基础设施建设。启动机场改扩建，开辟新航线，提高旅客吞吐量，争取航空货运实现重大突破。规划建设海港公共码头，扩大对外开放水域，建设产业枢纽港区。加强榕江航道整治，发展榕江港口产业群。建设梅汕客专揭阳段，加快揭阳疏港铁路、广梅汕铁路揭阳段改线和汕潮揭城际轻轨前期工作。推进潮惠、揭惠等高速公路建设，开工建设潮汕环线快速路、揭惠高速市区连接线等，加快国道206线、省道234线等路面改造工程，新（改）建地方公路500公里。

建设信息高速公路。建设“光网城市”，结合地下综合管廊建设，统筹网络管线布局升级。实施“满格揭阳”行动，支持无线电基站和公共无线局域网建设。引进中科曙光建设云计算中心，打造大数据枢纽基地。加强“三网融合”基础技术工程建设，推动广电、电信双向业务商用化。

提高物流通达效率。出台扶持政策，创建快递示范城市，推进快递物流园区建设，降低企业物流成本，鼓励快递分拨总部落户。推进农产品物流园、粤东农产品物流城等项目建设，引进发展冷链仓储配送。推广建设快递E站、快递智能箱。规范公交站点，整顿运输市场。建设综合保税仓，促进通关便利化。

（六）统筹抓好“三农”工作

支持农村业态创新。落实强农惠农政策，稳定粮食生产。培育新型农业经营主体，推进农业产业化经营。推进与省农业厅共建“一镇一品”示范区，培树金和镇互联网＋“一镇一品”示范区，支持揭西乡村旅游产业带、惠来农海产品电商港、普宁青梅电商产业园等探索创新。引导大中专毕业生、外出乡亲等返乡领办合作社和家庭农场，释放“双创”活力，促进农业与旅游、健康养老等深度融合。

夯实农业发展基础。扩大实施“沃土工程”，加大高标准农田和农业设施建设力度。完成榕江大围达标加固工程、市北河桥闸重建工程建设任务，加快邦山水闸重建工程等项目建设进度，增强防灾减灾和灌溉保障。加快惠来县中东部供水工程建设，启动韩江、龙颈水库引水工程，推进村村通自来水，确保饮水安全。建设神泉现代示范性渔港，实施标准渔港和标准鱼塘改造。以省级新农村示范片建设为重点，全面加强农村规划建设，强化村庄环境综合整治，推进美丽乡村建设。

深化农村综合改革。推进农村土地承包经营权确权登记颁证工作，引导农村土地承包经营权有序流转。深化农村集体产权制度改革，加强农村“三资”管理。设立现代农业发展基金，发展农业保险，开展农业补贴“三补合一”改革。推进农垦、供销社、国有林场和基层水管体制改革。培育和规范发展自然村（村民小组）村民理事会，加强村风建设，促进农村和谐稳定。

深入实施精准扶贫。突出产业扶贫导向，精准锁定扶贫对象，精准确定扶贫项目，着力解决贫困户就医就学、饮水安全、危房改造等问题，确保贫困人口脱贫。实施电商扶贫项目。动员社会力量参与扶贫，组织企业定向结对贫困村庄、贫困人口。

（七）竭力建设生态文明

推进环境综合整治。加强环保执法力度，深化打击重金属和纺织漂染等非法排污专项行动。全面落实“河长制”，推进榕江、练江、龙江综合整治和河涌清理。强力整治废气、扬尘和露天焚烧等大气污染源，净化空气环境。坚持最严格耕地保护制度，加大违建和闲置土地治理。加强海洋污染防治，建设美丽海湾。

加快生态工程建设。抓好9个国家、省责任书污水处理设施项目建设。启动达标排放纺织漂染园、酸洗产业园建设，抓好食品药品合作区治污先行工程。建设中德资源再生基地、第三代生活垃圾处理绿色示范工厂。加快普宁市垃圾焚烧发电厂和惠来县含尾坑垃圾填埋场建设，规范城市垃圾收集点，完善村镇生活垃圾收运体系和保洁清扫制度，力争75%以上农村生活垃圾得到有效处理。推进新一轮绿化揭阳大行动，突出抓好榕江、练江、龙江江畔植树绿化，建设三大绿色发展带。

培育发展环保产业。提升放大全国首家中德合作“零排放”电镀产业园的承载力、创造力、影响力，打造全要素、独具竞争力的绿色电镀产业集聚区，在金属产业国际分工中占据一席之地。依托欧绿保、安泰克等项目，建设先进环保设备生产基地，发展环保设备租赁服务。引入培育节能环保服务机构，推广合同能源管理，发展清洁生产和循环经济。

（八）协同促进各项事业

办好人民满意教育。巩固提高教育创强成果，启动创建教育现代化先进市。完成薄弱普通高中改造提升工程，办强办优揭阳一中。支持揭阳职业技术学院争创高水平学院，推动全市职业技术学校整合升级。推进与德国埃斯林根应用科技大学合作办学。发挥市职教联盟作用，推动电商培训机构向军埔村集聚，建设全国首个电商人力资源产业园区。推动每所职业院校对接1个以上行业协会，开展现代学徒制试点。鼓励青少年科技创新，培养创新型人才。联合北师大、华师大等高校推进“强师工程”，提高师资队伍素质。

繁荣发展文化事业。关爱扶持文化人才，推进基层文化设施建设，提升社区文化服务。加强爱国主义教育基地建设，推进揭阳学宫、丁日昌旧居修缮工程，守护好文化遗产。启用博物馆、群艺馆，建设文化人才创作基地，办好文化惠民品牌活动，强化公共文化流动服务。大力弘扬社会主义核心价值观，推动志愿者服务机制化、常态化。加快培育发展文化和体育产业，推进社区体育、文化公园建设和体育、文化场地设施开放。开展中德文化交流活动。

提升公众健康水平。深化医药卫生体制改革，完善多层次医疗卫生服务体系，建设卫生强市。落实卫生“强基创优”行动计划，创建互联网＋医疗示范医院。加快市人民医院、市慈云医院改扩建工程和普宁市新中医院建设，建设市中医院新住院大楼，改造提升县级人民医院，加强乡镇卫生院和村卫生站建设。鼓励社会资本办医，促进非公立医疗机构规范健康发展。健全传染病防控和救治机制，提升公共卫生服务。加强医疗卫生人才队伍建设，引进和培养全科医生，鼓励医生到基层多点执业。加强医德医风建设，构建和谐医患关系。联合专业保险机构，探索“互联网＋”政保医一体的新型健康医疗服务体系。落实“全面二孩”政策，转变计生服务方式，加强妇幼保健工作，提高出生人口素质。

强化就业保障。把就业扶持政策落到实处，促进就业新形态的形成和聚集，建设街区、镇村就业平台。加大技能培训力度，推动金融机构设计小额就业专项产品，多层次、高频率、多形式地开展双创激励活动。实施全民参保计划，加强社保扩面征缴，推动企业全员足额参保。完善社会救助体系，发展社会福利、慈善事业。支持老年教育，完善养老服务机构，发展养老产业。

加强财税金融工作。落实各项财税政策，促进税负公平，清理规范收费项目。优化财政支出结构，集中资源办大事、办实事、办可形成持续竞争力的事，重点促产业、保民生。以中德进出口贸易集聚区、揭西电线电缆回归工程和榕城后汽车产业街区为试点，探索税源集聚区培育新模式。以中德中小企业合作区为依托，引导金融机构、金融资源集聚，建设金融服务实体经济的创新平台。支持产业金融、普惠金融及互联网金融创新，支持企业上市，鼓励探索众筹众建的投融资模式，推进PPP建设试点。切实防范金融风险。

维护社会和谐稳定。提高城乡见警率，扎实抓好公安“四项”建设，创新完善立体化社会治安防控体系，依法严厉打击“两抢一盗”、“黄赌毒”、电信诈骗等违法犯罪行为，建设市消防应急救援训练基地及空港经济区特勤大队，推进全市社区戒毒、社区康复、禁毒教育基地建设，在惠来、普宁建设病残吸毒人员收治场所，强化安全生产、消防安全、食品药品安全和打假工作，打造平安揭阳。实施“七五”普法，完善“一村（社区）一法律顾问”制度，强化基层法治建设。依法推进信访维稳工作，化解社会矛盾纠纷，保障群众合法权益。

同时，推动军民融合发展，支持国防和军队改革。做好知识产权、新闻出版、广播电视、气象、地震、国防动员、民兵预备役、双拥、人防、应急、民族宗教、妇女儿童、红十字、社科、档案、方志、文史等工作。

（九）真切办好十件民生实事

1、实施幼儿园提升工程，新（改、扩）建63所公办幼儿园，指导推动全市民办幼儿园上水平。学前教育家庭经济困难幼儿生活费补助标准从每生每年300元提高到1000元。

2、探索互联网＋医疗，实现每县（市、区）建立一所以上互联网＋试验医院，全市县级以上医院实现与上级医院远程会诊，带动两所以上镇级医院开展远程会诊；建成榕城、揭西2个县级出生缺陷综合干预中心，为符合政策育龄夫妇提供免费优生健康检查。

3、建成营运9家污水处理厂，在练江、榕江流域推广建设小区域污水处理中心，完成榕江8条来水综合治理。

4、统筹3000万元投入市区环卫事业，强化环卫队伍建设管理，配齐扫路车、垃圾压缩车、抑尘车等现代化环卫机械，提升市区保洁水平。

5、启动古城改造工程，完成城市棚户区改造2972户和省下达农村危房改造任务。

6、建设及提升黄岐山、东湖、西湖、中德贝多芬、北河桥头、空港新区、蓝城体育公园等七大公园，实施社区公园、社区绿地规划建设专项计划。

7、实现重点公共场所免费WIFI网络全覆盖，全市100%行政村光纤全覆盖，全市农村电商服务站全覆盖。

8、全面优化市区公交线路，新增新能源公交车200辆。

9、全市新增辅警300名；新增城乡高清探头500个，在市际和重点场所布建电子围栏30个；成立市见义勇为基金。

10、建成粤东（揭阳）残疾人集中就业培训中心，实施残疾人“展翅”双创工程。

（十）全面加强政府自身建设

深化行政改革。深入转变政府职能，进一步简政放权，降低企业制度性交易成本，营造市场化国际化法治化营商环境。深化行政审批制度和商事制度改革，继续压缩审批项目和环节，全面推行网上审批。改进执法方式，强化重点领域监管，维护市场经营秩序。推进综合信用体系建设。推进电子政务建设，加强政府信息公开，让权力在阳光下运行。

强化依法行政。自觉接受人大、政协和社会各界监督，办好人大议案建议和政协提案。深入贯彻《法治政府建设实施纲要（2015－2020年）》，推动制定地方性法规和政府规章，加快依法治市进程。健全重大行政处罚备案审查制度，加强行政复议工作。建立健全政府法律顾问制度，组建法律顾问团队。为县（市、区）长和市直局长配备专家顾问，发挥专家决策咨询作用，完善政府科学决策程序。

建设廉洁政府。巩固扩大党的群众路线和“三严三实”教育实践活动成果，深入贯彻落实中央“八项规定”、国务院“约法三章”精神，坚持厉行节约、倡俭治奢，持之以恒纠正“四风”。严格落实党风廉政建设责任制，抓好政府系统党风廉政教育和公务员队伍建设，完善监察、司法、审计等联动的行政权力监督机制。强化督促检查和督查问责，严厉查处为官不为等失职渎职行为，全面整治庸政懒政怠政，促进干部清正、政府清廉、政治清明，以好的政风促社风民风。

各位代表！让我们紧密团结在以习近平同志为总书记的党中央周围，在省委省政府和市委的坚强领导下，真抓实干，努力开创揭阳振兴发展新局面，为实现中华民族伟大复兴的“中国梦”和我省“三个定位、两个率先”目标作出更大贡献！
